# Supplementary material for: In vitro evaluation of Escherichia coli and Staphylococcus aureus translocation in 3D printed material
Source: bioRxiv. 2026 Feb 2:2026.02.02.703277. Preprint. [Version 1] doi: 10.64898/2026.02.02.703277 (PMC12889644; doi:10.64898/2026.02.02.703277)
Supplement: Supplement 1 [file media-1.pdf]

Supplementary Information

Title: *In vitro* evaluation of *Escherichia coli* and *Staphylococcus aureus* translocation in 3D printed material

Authors: Ashma Sharma[1a], Joshua Prince[1b], A-Andrew D Jones III[1234c*]

Affiliations:

1. Department of Civil & Environmental Engineering, Pratt School of Engineering, Duke University, Durham, NC 27708
2. Thomas Lord Department of Mechanical Engineering & Materials Science, Pratt School of Engineering, Duke University, Durham, NC 27708
3. Duke Microbiome Initiative, Duke University, Durham, NC 27708
4. Duke Materials Initiative, Duke University, Durham, NC 27708
    a. Ashmasharma5@gmail.com: ORCID: 0000-0002-4482-9449
    b. Present address: ICF International ORCID: 0000-0001-6050-9544
    c. andrew.jones3@duke.edu, ORCID: 0000-0003-3840-8039

*Corresponding Author

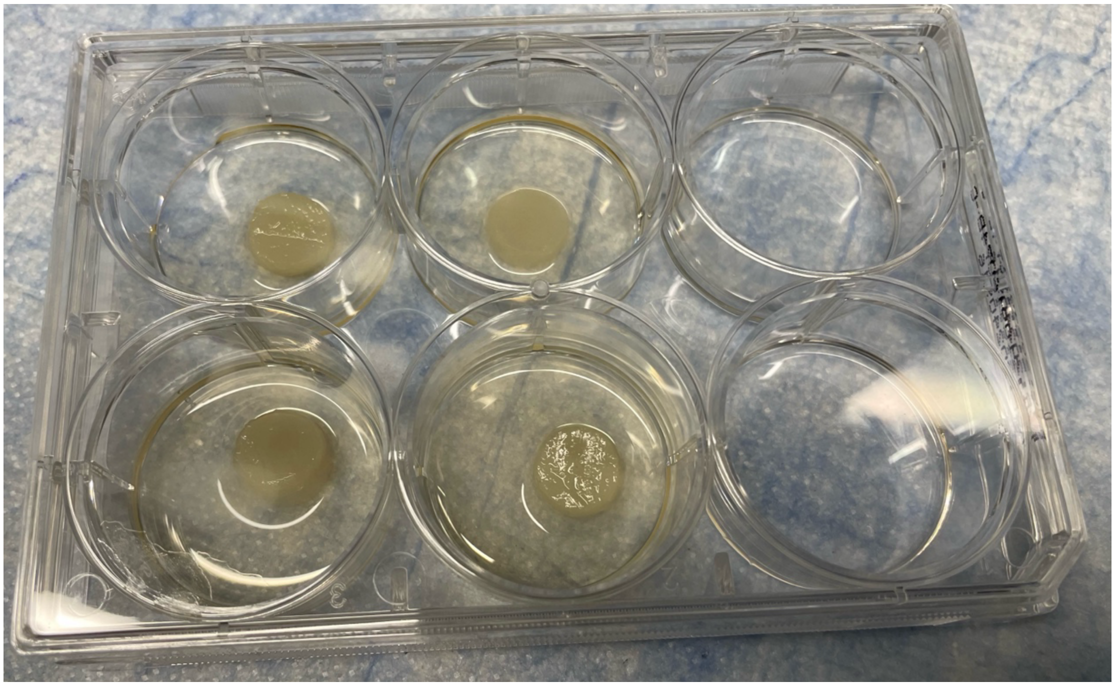

SI Figure 1: Culturing of bacteria against construct in 6-well plates. The media is filled to the midline of the biomaterial construct.

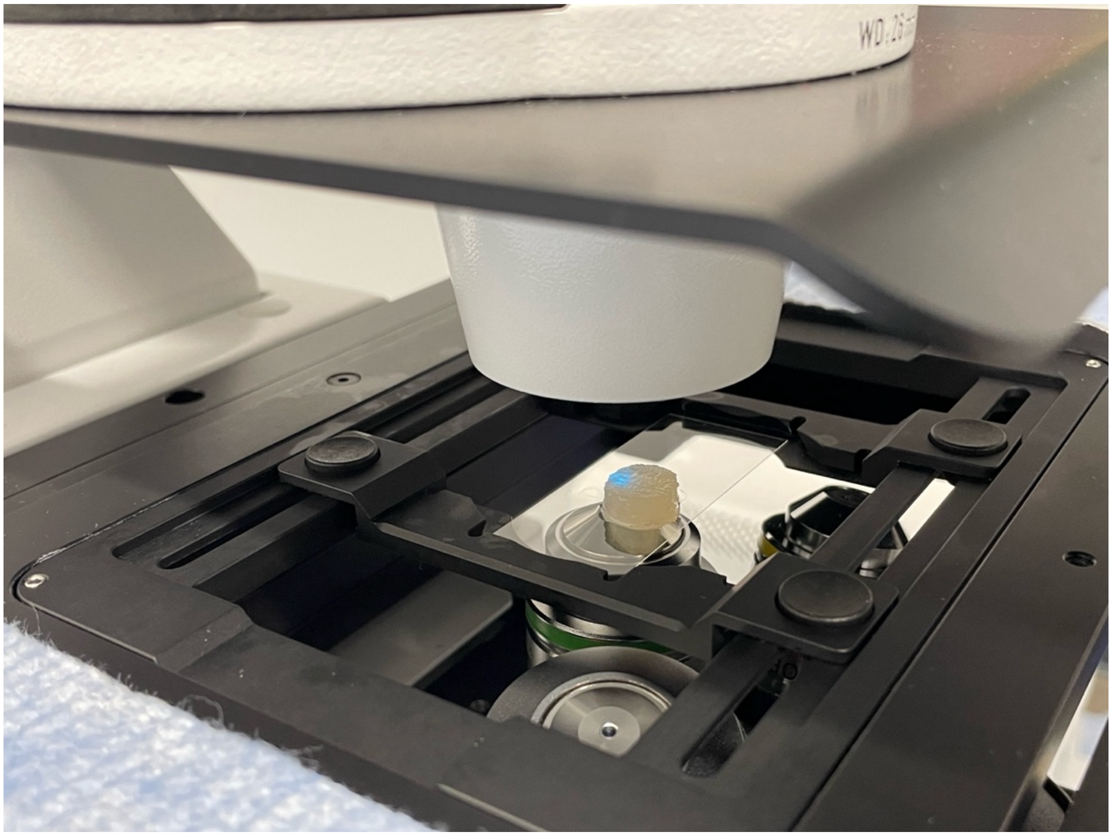

SI Figure 2. Set up of the confocal microscopy of each construct. Glass slides were used to balance the construct and images were taken using an inverted microscope.

SI Table S1. ***E. coli*** **and** ***S. aureus*** **penetration into alginate, GelMA, and porcine tissue mean and 95% confidence interval statistics shown for averages of technical replicates with bootstrapping from Figure 5.**

Material	Species	Biological Replicate Average	Biological Replicate SD	Biological Replicate 95%CI Magnitude	Time [hrs]
Tissue	*E. coli*	2246.375	368.910	361.526	2
Tissue	*E. coli*	2226.417	37.659	36.905	4
Tissue	*E. coli*	2162.875	641.807	628.959	6
GelMA	*E. coli*	1615.667	54.212	61.346	2
GelMA	*E. coli*	1797.667	32.194	36.431	4
GelMA	*E. coli*	1607.778	84.555	95.681	6
Alginate	*E. coli*	1685.778	43.900	49.677	2
Alginate	*E. coli*	1648.889	118.140	133.686	4
Alginate	*E. coli*	1676.556	67.841	76.768	6
Tissue	*S. aureus*	1955.667	135.179	132.473	2

Tissue	*S. aureus*	1894.083	432.366	423.711	4
Tissue	*S. aureus*	2334.667	45.352	44.444	6
GelMA	*S. aureus*	2327.744	53.558	60.605	2
GelMA	*S. aureus*	2306.778	105.998	119.946	4
GelMA	*S. aureus*	2207.111	61.800	69.932	6
Alginate	*S. aureus*	2053.011	110.957	125.558	2
Alginate	*S. aureus*	1969.000	363.284	411.086	4
Alginate	*S. aureus*	1955.667	141.720	160.368	6

SI Table 2. Breakthrough rates represented by averages of three technical replicates for three biological replicates in Log CFU/mL of *E. coli* and *S. aureus* from Figure 6. One-way ANOVA Tukey multiple comparisons of means 95% family-wise confidence level.

Material	Species	Biological Replicate Average	Biological Replicate SD	Biological Replicate 95%CI Magnitude	Time [hrs]
Tissue	*E. coli*	4.996	1.462	3.632	2
Tissue	*E. coli*	1.963	3.400	8.446	4
Tissue	*E. coli*	2.398	4.153	10.316	6
PC	*E. coli*	6.066	0.040	0.099	2
PC	*E. coli*	7.088	0.063	0.156	4
PC	*E. coli*	7.515	0.105	0.261	6
GelMA	*E. coli*	6.486	0.282	0.701	2
GelMA	*E. coli*	7.125	0.207	0.513	4
GelMA	*E. coli*	7.685	0.124	0.309	6
Alginate	*E. coli*	6.160	0.055	0.136	2
Alginate	*E. coli*	7.321	0.543	1.348	4
Alginate	*E. coli*	7.799	0.030	0.075	6
Tissue	*S. aureus*	5.138	0.652	1.618	2
Tissue	*S. aureus*	5.882	1.171	2.909	4
Tissue	*S. aureus*	5.612	2.044	5.078	6
PC	*S. aureus*	4.360	0.219	0.544	2
PC	*S. aureus*	5.452	0.414	1.029	4
PC	*S. aureus*	6.119	0.144	0.357	6
GelMA	*S. aureus*	6.418	0.102	0.252	2
GelMA	*S. aureus*	7.951	0.067	0.166	4
GelMA	*S. aureus*	8.142	0.110	0.273	6
Alginate	*S. aureus*	6.175	0.029	0.072	2
Alginate	*S. aureus*	6.108	0.010	0.024	4

Alginate	*S. aureus*	7.328	0.024	0.059	6
